# Comprehensive Analysis of Immune Implications and Prognostic Value of SPI1 in Gastric Cancer

**DOI:** 10.3389/fonc.2022.820568

**Published:** 2022-02-14

**Authors:** Jianfeng Huang, Wenzheng Chen, Zhigang Jie, Mengmeng Jiang

**Affiliations:** ^1^Department of Gastrointestinal Surgery, The First Affiliated Hospital of Nanchang University, Nanchang, China; ^2^Department of Emergency Surgery, The First Affiliated Hospital of Nanchang University, Nanchang, China

**Keywords:** gastric cancer, SPI1, prognostic markers, risk signature, nomogram

## Abstract

**Background:**

The transcription factor Spi-1 proto-oncogene (SPI1, also known as PU.1) is a key regulator of signal communication in the immune system and is essential for the development of myeloid cells and lymphocytes. However, the potential role of SPI1 in gastric cancer (GC) and the correlations between SPI1 and immune infiltration remain unclear.

**Methods:**

In the present study, multiple databases including ONCOMINE, TIMER, Kaplan–Meier Plotter, and The Cancer Genome Atlas were used to explore the expression levels and prognostic value of *SPI1* in GC. cBioPortal was used to explore the possible reasons for the increased expression of SPI1 in GC. The correlations between SPI1 expression and tumor-infiltrating immune cells (TICs) were analyzed using CIBERSORT and TIMER. Gene set enrichment analysis was used to determine the biological function of SPI1 in the development of GC. In addition, a risk signature based on SPI1-related immunomodulators was constructed to accurately evaluate the prognosis of patients with GC. The upregulation of SPI1 expression in GC was further confirmed through immunohistochemistry, western blotting, and real-time quantitative PCR (RT-qPCR) assay.

**Results:**

The expression of SPI1 was increased significantly in GC according to multiple databases, and high expression of SPI1 was related to poor prognosis and progression of GC. The main factor influencing the high expression of SPI1 mRNA in GC may be diploidy, not DNA methylation. Moreover, immunohistochemistry, western blotting, and RT-qPCR assays also confirmed the upregulated expression of SPI1 in GC. CIBERSORT analysis revealed that SPI1 expression was correlated with seven types of TICs (naive B cells, resting memory CD4 T cells, activated memory CD4 T cells, activated natural killer cells, resting natural killer cells, M2 macrophages, and resting dendritic cells). Gene set enrichment analysis indicated that SPI1 might be related to immune activation in GC and participate in cell cycle regulation. In addition, based on SPI1-related immunomodulators, we developed multiple-gene risk prediction signatures and constructed a nomogram that can independently predict the clinical outcome of GC.

**Conclusion:**

The results of the present study suggest that SPI1 has a critical role in determining the prognosis of GC patients and may be a potential immunotherapeutic target.

## Introduction

In recent years, gastric cancer (GC) has become the fifth most common cancer worldwide and ranks third among cancer-related deaths (1). The introduction of standardized treatment, including surgery, chemotherapy, and targeted therapy, has improved the 5-year survival rate of patients with GC; however, it outcomes are still unsatisfactory (2). Late tumor-node-metastasis (TNM) stage, poor differentiation, and ineffective treatment targets are the key factors leading to tumor recurrence and metastasis (3, 4). Therefore, there is an urgent need to explore the molecular mechanisms underlying the occurrence and development of GC, and to identify novel drug therapy targets.

Spi-1 proto-oncogene (SPI1, also known as PU.1), which was first isolated by Moreau-Gcherin et al. (5) from Friend hemoglobin disease, is an erythroblast transformation-specific (ETS) family transcription factor. The proto-oncogene *SPI1* is located in the p11.22 region of human chromosome 11 (6). The SPI1 protein is homologous to SPI-B and SPI-C and is located in the nucleoplasm (7). SPI1 is essential for the development of myeloid cells and lymphocytes, and defects in or knockout of *SPI1* can lead to defects in granulocytes, monocytes, macrophages, and B cells (8, 9). In addition, the decreased expression of *SPI1* caused by heterozygous deletion of the *SPI1* locus eventually leads to the occurrence of acute myeloid leukemia (AML) (10). Previous studies have found that SPI1 acts as a tumor suppressor by regulating cell cycle and apoptosis in classical Hodgkin’s lymphoma cells (11). Tschan et al. also found that SPI1 could impair the transcriptional activity of SPI1 by binding to specific regions of P53, thereby regulating the cell cycle and apoptosis (12). In recent years, research has shown that SPI1 might be related to the progression of breast cancer (13), cervical cancer (14), lung cancer (15), glioma (16), and other malignant tumors.

Both endogenous heredity and the exogenous tumor microenvironment (TME) are significant factors that affect the occurrence and development of GC (17). When the immune effector cells in the TME reach a certain balance, immune activation occurs, which facilitates the immune escape of tumor cells (18, 19). Tumor infiltration by tumor-associated macrophages, dendritic cells, and regulatory T cells is associated with poor prognosis in GC (20, 21). However, high expression of CD3, CD8, and CD45RO in tumor-infiltrating lymphocytes was associated with longer survival of GC patients (22). Many studies have shown that immune infiltration induced by immune signaling is closely related to prognosis in GC, and tumor-infiltrating immune cells (TICs) could represent an important breakthrough in the treatment of GC. The transcription factor SPI1, as a key regulator of signal communication in the immune system (23), may be related to the prognosis of GC. However, the mechanism by which SPI1 participates GC progression and immune infiltration remains unclear.

We first found that the expression of SPI1 is upregulated in GC through Oncomine, TIMER, Kaplan–Meier plotter and TCGA database, and it is associated with poor prognosis. Second, through CIBERSORT, Gene Ontology (GO) and the Kyoto Encyclopedia of Genes and Genomes (KEGG) analysis, we found that SPI1 is related to a variety of TICs and tumor-related signaling pathways. In addition, we developed a risk signature based on SPI1-related immunomodulators and constructed a nomogram that can independently predict the clinical outcome of GC. Finally, we confirmed the up-regulated expression of SPI1 in GC through immunohistochemistry, western blotting and RT-qPCR analysis. Overall, our results indicated that SPI1 can act as a novel prognostic biomarker and provide new ideas GC immunotherapy.

## Materials and Methods

### ONCOMINE, TIMER, and The Cancer Genome Atlas (TCGA) Analysis

Using the online cancer database ONCOMINE (https://www.oncomine.org/) (24) TIMER (https://cistrome.shinyapps.io/timer/) (25), a database of cancer immune-infiltration levels, we analyzed the transcription levels of *SPI1* in various cancer tissues. We then use the data set in the TCGA database to verify the results *via* pairing analysis. The RNA sequencing data and clinical data of 407 patients with stomach adenocarcinoma (STAD) (32 normal specimens and 375 tumor specimens) were downloaded from the TCGA database (https://portal.gdc.cancer.gov/).

### Kaplan–Meier Plotter Database Analysis

Kaplan–Meier Plotter uses many cancer samples to assess the effect of a gene on the survival of patients with a particular cancer. These include samples from breast cancer, ovarian cancer, lung cancer, stomach cancer, and liver cancer from different sources. The correlation between *SPI1* expression and survival in GC was analyzed using Kaplan–Meier Plotter (http://kmplot.com/) (26).

### CBioPortal Database Analysis

The public database cBioPortal (https://www.cbioportal.org/) (27) contains a variety of data that can be used to analyze, for example, genetic alterations, copy number variations, and abnormal methylation. Through cBioPortal, we systematically analyzed the genetic alterations of SPI1 in different types of GC and the effect of copy number changes on the expression level of SPI1.

### TICs Profile

The TIC abundance distribution of all tumor samples was estimated using the CIBERSORT computational method, and then 230 tumor samples with P < 0.05 were selected for the subsequent analysis.

### GO and KEGG Enrichment Analysis

To explore the biological function of SPI1 in GC, we used GO and KEGG enrichment analysis with the Gene Set Enrichment Analysis (GSEA) software and the R language. Only p and q < 0.05 were regarded to indicate significant enrichment.

### Identification and Validation of SPI1-Related Immunomodulators

We used online integrated database TISIDB (http://cis.hku.hk/TISIDB/) (28) to retrieve immunomodulators associated with SPI1. We chose immunoinhibitors and immunostimulators that were significantly correlated with SPI1 with respect to gene expression (Spearman correlation test, P<0.05, r >0.6). Next, we performed univariate Cox regression analysis to obtain immunomodulators related to the survival of patients with GC. To further establish a risk signature of immunomodulators related to SPI1, stepwise variable selection was performed using the Akaike information criterion in the Cox models. After the immunomodulators had been chosen, the prognostic index, referred to as the risk score, was generated as follows: risk score = ∑ (Coefi * Expxi), where Expxi represents the expression level of each gene and Coefi represents the coefficient of each gene. Kaplan–Meier and receiver operating characteristic (ROC) curves were used to evaluate the performance of the risk signature. Univariate and multivariate Cox regression analyses between the risk signature and clinicopathological features were performed, and P < 0.05 was considered to indicate a significant independent prognostic factor.

### Construction of a Nomogram

The nomogram was constructed based on independent clinical factors including risk score, age, gender, grade, stage, T, N and M by using the “regplot” R package to quantify risk evaluation and predict the clinical outcomes of patients with GC. The ROC curve and the calibration plot were applied to assess the accuracy of the nomogram for predicting the 1-, 2-, and 3-year overall survival of patients with GC.

### Cell Lines and Culture

Three GC cell lines including MGC-803, HGC-27, AGS and the human normal gastric epithelial cell line were obtained from American Type Culture Collection (ATCC, USA) or Shanghai Cell Bank (Shanghai, China). These cell lines were cultured at 5% CO2 and 37°C in a humidified atmosphere as recommended.

### Immunohistochemistry

Tissue samples were paraffin embedded, sliced, and used for immunohistochemical testing according to standard procedures. Antibodies against SPI1 (1:100, ab76543, Abcam, UK) were used.

### Western Blot Analysis

The protein levels of SPI1 in both cell lines and fresh human tissues were detected using western blotting. In short, 42 mg of protein was separated by a 10% SDS-PAGE gel and then transferred to a PVDF membrane (Millipore, USA). The membrane was blocked with 5% BSA for 1 hour at room temperature. Next, the membrane was incubated with SPI1 antibody (1:1000, ab76543, Abcam, UK) overnight at 4°C. The membrane was then incubated with the secondary antibody for 1 hour at room temperature. The protein bands were visualized with a chemiluminescence solution. Tubulin was used as a control.

### RT-qPCR

The mRNA levels of SPI1 were examined using RT quantitative PCR (RT-qPCR). Total RNA was extracted by trizol reagent according to the manufacturer’s protocol. RNA was reverse-transcribed into cDNA and subjected to PCR reactions using the EasyScript One-Step RT-PCR kit (TRAN, AE311-03). GAPDH was used as control. The primers used in this study were as follows: SPI1 (F: ATGAAGGACAGCATCTGGTGG, R: TTCACCTTCTTGACCTCGCCC) GAPDH (F: CTGCCTCTACTGGCGCTG, R: GGTCAGGTCCACCACTGAC). The relative expression of SPI1 mRNA was normalized to the expression level of mRNA using the 2−ΔCt method.

### Statistical Analysis

The R software and Perl language were used for all statistical analyses. Expression data were normalized by log2 transformation. The logarithmic rank test was used in the survival analysis. Spearman’s correlation test was used for the correlation analysis. P < 0.05 was considered to indicate statistical significance.

## Results

### Detection of Aberrant Expression of SPI1 mRNA in Human Cancers *via* Bioinformatics Analyses

**Figure 1:**
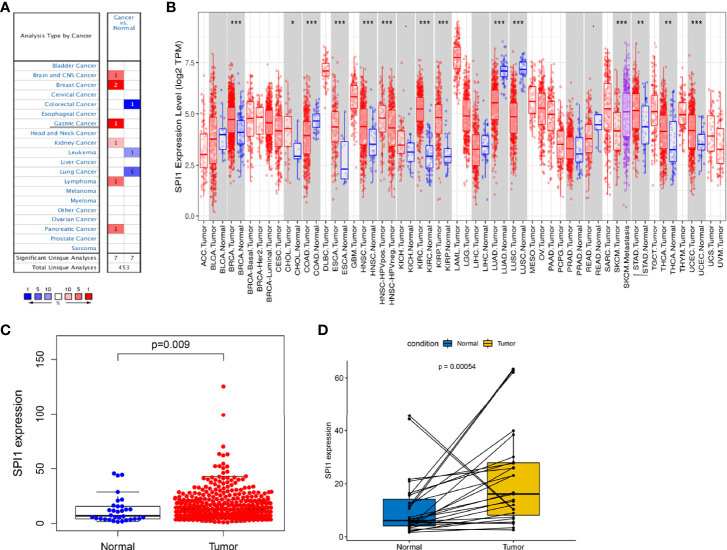
SPI1 expression levels in different types of cancers. **(A)** Expression of SPI1 in different cancer tissues increased or decreased. **(B)** Human SPI1 expression levels in multiple cancers determined using TIMER analysis. **(C)** Differential expression of *SPI1* in normal gastric tissue and gastric cancer tissue. **(D)** Paired differential analysis for the expression of *SPI1* in normal and tumor samples derived from the same patient. *P < 0.05, **P < 0.01, ***P < 0.001.

To compare the mRNA expression levels of *SPI1* in normal and tumor tissues among multiple human cancer types, we used the ONCOMINE and TIMER databases. Compared with that in normal tissues, *SPI1* expression was higher in brain cancer, head and neck cancer, breast cancer, GC, kidney cancer, pancreatic cancer, and lymphoma; and lower in colorectal cancer, lung cancer, and leukemia (**Figures 1A, B**). To further evaluate *SPI1* expression in GC, we downloaded a dataset of 375 GC samples and 32 normal samples (including RNA sequencing and clinical information) from the TCGA database. The results showed that the expression of *SPI1* in GC tissues was significantly higher than that in normal samples (**Figure 1C**). This result was also fully verified in a paired analysis of GC tissue and adjacent normal tissue in the same patient (**Figure 1D**).

### High Expression of SPI1 Predicts Poor Prognosis in Patients With GC

**Figure 2:**
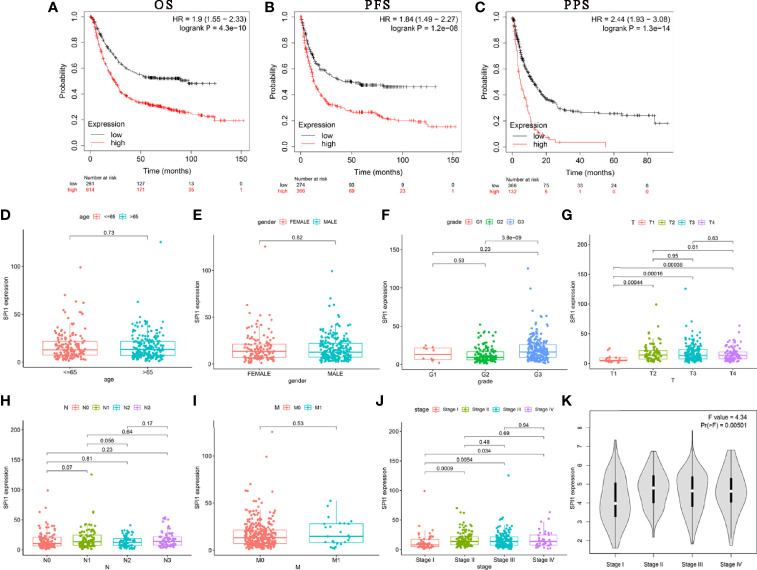
SPI1 expression is associated with poor survival in GC patients. **(A–C)** OS, PFS, and PPS survival curves for GC patients. Correlations between *SPI1* expression and clinicopathological features. The clinicopathological features of STAD included **(D)** age, **(E)** sex, **(F)** tumor differentiation grade, **(G)** T stage, **(H)** N stage, **(I)** M stage, and **(J, K)** TNM stage.

Next, we used Kaplan–Meier Plotter to analyze whether the expression of *SPI1* was related to the prognosis of patients with cancer and the extent to which it was related. Interestingly, we identified that poor prognosis of GC was correlated with higher SPI1 expression: overall survival (OS) hazard ratio (HR) = 1.9, 95% confidence interval (CI) = 1.55 to 2.33, P = 4.3e-10; progression free survival (PFS) HR = 1.84, 95% CI = 1.49 to 2.27, P = 1.2e-08; post-progression survival (PPS) HR = 2.44, 95% CI = 1.93 to 3.08, P = 1.3e-14) (**Figures 2A–C**). In addition, we further examined the correlation of SPI1 with the clinicopathologic features of GC. Overexpression of SPI1 was related to worse OS, PFS, and PPS in patients grouped by sex, T stage, N stage, M stage, clinical stages, and Lauren classification (P < 0.05) but was not associated with differentiation (P > 0.05) (**Table 1**). In addition, high SPI1 expression had the highest HR values of clinical stage 1 of OS, PFS, and PPS in the four clinical stage categories (OS HR = 3.07; PFS HR = 1.75; PPS HR = 3.6). However, another set of data from the TCGA cohort indicated that *SPI1* expression was correlated closely with T stage and clinical stage of patients with GC (P < 0.05) but not with other clinical features (**Figures 2D–K**). The above results indicate that the expression of *SPI1* could be used as a potential diagnostic and prognostic marker of GC, with high expression indicating poor prognosis.

**Table 1:** Correlations between *SPI1* mRNA expression and clinical prognosis in gastric cancer with different clinicopathological factors using Kaplan–Meier analysis.

Clinicopathological characteristics	Overall survival (n = 881)			Progression-free survival (n = 645)			Post progression survival (n = 503)		
	N	Hazard ratio	P-value	N	Hazard ratio	P-value	N	Hazard ratio	P-value
**Sex**									
Female	236	2.15 (1.49–3.1)	**2.6e–05**	201	2.04 (1.4–2.97)	**0.00016**	149	2.56 (1.68–3.91)	**6.7e–06**
Male	544	1.9 (1.52–2.39)	**1.8e–08**	437	1.89 (1.45–2.47)	**2.3e–06**	348	2.48 (1.88–3.28)	**3.5e–11**
**Stage**									
1	67	3.07 (1.14–8.21)	**0.019**	60	1.75 (0.58–5.28)	0.31	31	3.6 (0.69–18.91)	0.11
2	140	2.00 (1.09–3.66)	**0.022**	131	1.95 (1.05–3.64)	**0.032**	105	1.89 (0.98–3.65)	0.054
3	305	1.81 (1.33–2.45)	**0.00011**	186	1.69 (1.16–2.46)	**0.0059**	142	2.65 (1.71–4.1)	**5.4e–06**
4	148	1.75 (1.15–2.64)	**0.0076**	141	1.54 (1.02–2.32)	**0.037**	104	1.7 (1.07–2.68)	**0.022**
**Stage T**									
2	241	1.69 (1.11–2.59)	**0.013**	239	1.7 (1.13–2.57)	**0.011**	196	1.76 (1.13–2.74)	**0.012**
3	204	1.64 (1.16–2.33)	**0.0047**	204	1.42 (1.02–2)	**0.039**	150	2.19 (1.46–3.28)	**9.2e–05**
4	38	2.18 (0.85–5.55)	0.095	39	2.69 (1.16–6.24)	**0.017**	29	0.33 (0.09–1.18)	0.074
**Stage N**									
0	74	1.56 (0.62–3.88)	0.34	72	1.51 (0.61–3.74)	0.37	41	6.28 (0.8–49.23)	**0.046**
1	225	2.61 (1.73–3.94)	**2.1e–06**	222	2.38 (1.61–3.52)	**7.9e–06**	169	3.49 (2.21–5.52)	**1.3e–08**
2	121	2.09 (1.29–3.37)	**0.0021**	125	1.77 (1.15–2.74)	**0.0087**	105	2.38 (1.43–3.98)	**0.00063**
3	76	1.92 (1.09–3.35)	**0.021**	76	1.75 (1.01–3.04)	**0.045**	63	1.82 (0.99–3.36)	**0.05**
1+2+3	422	1.86 (1.41–2.44)	**6.2e–06**	423	1.7 (1.31–2.22)	**5.8e–05**	337	2.23 (1.68–2.96)	**1.5e–08**
**Stage M**									
0	444	1.71 (1.28–2.27)	**2e–04**	443	1.58 (1.21–2.08)	**8e–04**	342	2.18 (1.61–2.94)	**1.8e–07**
1	56	2.51 (1.25–5.03)	**0.0077**	56	2.05 (1.02–4.13)	**0.041**	36	3.98 (1.66–9.55)	**0.00093**
**Lauren classification**									
Intestinal	320	2.28 (1.65–3.17)	**3.9e–07**	263	2.18 (1.36–3.49)	**0.00087**	192	2.66 (1.76–4.02)	**1.5e–06**
Diffuse	241	1.64 (1.16–2.3)	**0.0044**	231	1.63 (1.14–2.35)	**0.0075**	176	1.74 (1.18–2.58)	**0.0046**
Mixed	32	1.89 (0.65–5.51)	0.24	28	0.22 (0.06–0.82)	**0.014**			
**Differentiation**									
Poorly	165	0.68 (0.44–1.03)	0.067	121	0.62 (0.39–0.98)	**0.038**	49	1.44 (0.76–2.73)	0.26
Moderately	67	1.76 (0.92–3.36)	0.085	67	1.71 (0.92–3.2)	0.088	24	0.57 (0.2–1.61)	0.28
Well	32	2.42 (0.81–7.21)	0.1						

Names of clinicopathological features and significant P-values are in bold.

### Genomic Alterations of *SPI1* in GC

**Figure 3:**
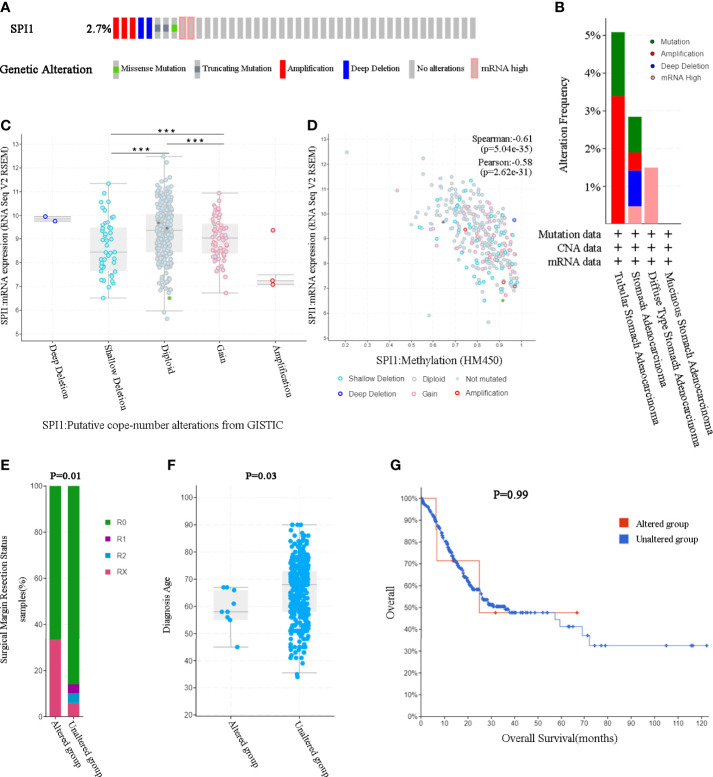
*SPI1* genomic alterations in GC (cBioPortal). **(A)** OncoPrint of *SPI1* alterations in the GC cohort. **(B)** The frequency and type of *SPI1* genetic alterations in different types of GC. **(C)** *SPI1* expression in different *SPI1* CNA groups. **(D)** Relationship between the methylation level (HM450) and the mRNA expression of *SPI1*. **(E, F)** Compared with the unaltered group, the group with *SPI1* gene alterations was related to surgical margin resection status and diagnosis age. **(G)** The log-rank test was used to analyze the relationship between SPI1 genetic alterations and the overall survival time of patients with GC (P = 0.99). ***P < 0.001.

Considering that genomic alterations usually lead to changes in gene expression levels and even related diseases, we used the cBioPortal tool to comprehensively analyze the *SPI1* alterations in STAD. *SPI1* was altered in 10 of 369 (2.7%) patients with STAD (**Figure 3A**). These alterations included mRNA upregulation, amplification, mutation, and deletion. We then analyzed the type and frequency of genomic alterations in different types of gastric adenocarcinoma (**Figure 3B**). *SPI1* diploidy resulted in high expression levels of *SPI1* in STAD. Compared with the gain group and shallow deletion group, the diploid group had the highest *SPI1* expression levels (P < 0.001) (**Figure 3C**). However, as shown in **Figure 3D**, the expression level of *SPI1* was correlated negatively with the *SPI1* methylation level (HM450) in different copy number alteration (CNA) groups. We then further explored whether *SPI1* genomic alterations affected the clinical characteristics and survival of patients with GC. We found that the rate of positive surgical margin in the group with altered *SPI1* was higher than that in the unaltered group, and the age of initial diagnosis was younger (P < 0.05) (**Figures 3E, F**). Furthermore, a Kaplan–Meier plot and log-rank test demonstrated no significant effect of *SPI1* genetic alterations on the OS of patients with GC (**Figure 3G**).

### Associations of SPI1 With TICs in GC

**Figure 4:**
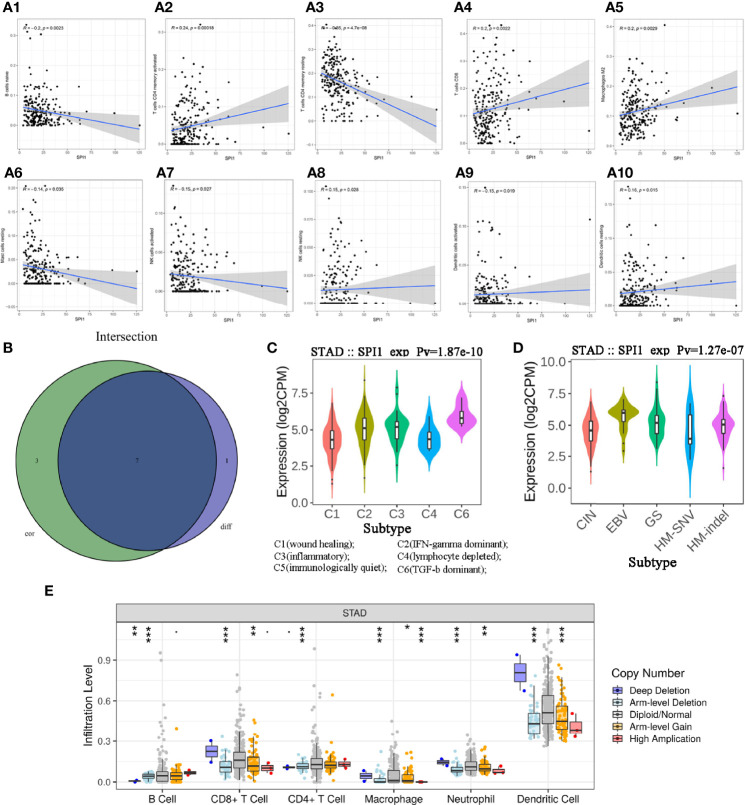
Correlation between SPI1 expression level and TICs in GC. **(A)** Scatter plot showing 10 types of immune cells correlated positively or negatively with the expression of SPI1. **(B)** Venn diagram showing seven types of immune cells correlated with SPI1 expression codetermined by difference and correlation tests, shown in violin and scatter plots, respectively. **(C, D)** Expression of SPI1 in different immune subtypes and molecular subtypes of GC. **(E)** CNA of SPI1 was related significantly to the immune infiltration levels of several immune cell types in GC, as assessed by TIMER analysis. *P < 0.05, **P < 0.01, ***P < 0.001.

To investigate the effects of *SPI1* expression on the immune microenvironment in GC, we used the CIBERSORT algorithm to determine the proportion of TICs (**Supplementary Figure 1A**) and constructed profiles for 22 types of immune cells in GC samples. The resulting correlation matrix revealed that CD8 T cells had the strongest positive correlation with activated memory CD4 T cells. However, CD8 T cells had a negative correlation with resting memory CD4 T cells and M0 macrophages (**Supplementary Figure 1B**). In addition, the Wilcoxon rank-sum test was used to accurately compare the difference, and showed that activated memory CD4 T cells, resting natural killer (NK) cells, M2 macrophages, resting dendritic cells, and neutrophils were more enriched in the *SPI1* high expression group than in the *SPI1* low expression group; however, naive B cells, resting memory CD4 T cells, and activated NK cells were enriched in the *SPI1* low expression group (**Supplementary Figure 1C**). The results of the correlation analyses showed that CD8 T cells, activated memory CD4 T cells, resting NK cells, M2 macrophages, and resting dendritic cells were correlated positively with *SPI1* expression; however, naive B cells, resting memory CD4 T cells, activated NK cells, activated dendritic cells and resting mast cells were correlated negatively with *SPI1* expression (**Figure 4A**). Based on the results of the difference and correlation analyses, we concluded that SPI1 might regulate the immune activity of the TME in GC primarily through seven types of TICs (**Figure 4B**).

### Potential of SPI1 in Immune Therapy

TISIDB was used to further understand the role of SPI1 and other biomarkers in the immunotherapy of GC. After dividing GC samples into wound healing, interferon (IFN)-gamma dominant, inflammatory, lymphocyte depleted, immunologically quiet and transforming growth factor beta (TGF-β) dominant groups, the results showed that *SPI1* expression in the TGF-β dominant group was higher than that in the other groups, whereas *SPI1* expression in the wound healing group was lower than that in the other groups (**Figure 4C**). As shown in **Figure 4D**, after dividing GC samples into five groups (CIN, EBV, GS, HM-SNV, HM-indel), *SPI1* expression was observed to be lowest in the HM-SNV subtype and highest in the EBV subtype. In addition, we also found that CNAS of *SPI1* greatly affected the degree of immune cell infiltration in GC (**Figure 4E**).

### Gene Set Enrichment Analysis of SPI1-Related Functional Networks

**Figure 5:**
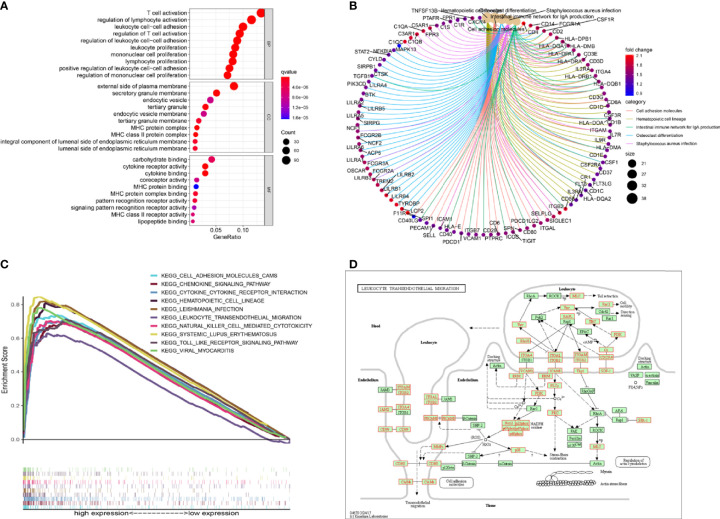
Gene set enrichment analysis. **(A, B)** GO and KEGG enrichment analyses of SPI1. **(C)** Multiple pathways in the KEGG analysis were related to high SPI1 expression. **(D)** KEGG pathway annotations of leukocyte transendothelial migration.

To better understand the underlying mechanisms associated with SPI1 in GC, gene ontology (GO) and Kyoto Encyclopedia of Genes and Genomes (KEGG) analyses were performed. The results of the GO enrichment analysis indicated that the main functions of SPI1 correspond to immune-related activities (**Figure 5A**). The results of the KEGG signaling pathway analysis are shown in **Figure 5B**. The three significantly enriched KEGG pathways were leukocyte transendothelial migration (**Figure 5D**), cell adhesion molecules (**Supplementary Figure 2**), and cell cycle (**Supplementary Figure 3**). As shown in **Figure 5C**, high expression of SPI1 is enriched for a variety of immune function gene sets. However, for low expression of SPI1, few immunological gene sets were enriched. This suggests that that SPI1 might be a key indicator that reflects the state of the TME and regulates immune activity, and that it might be involved in the regulation of the cell cycle.

### Prognostic Implications of SPI1-Related Immunomodulators in GC

**Figure 6:**
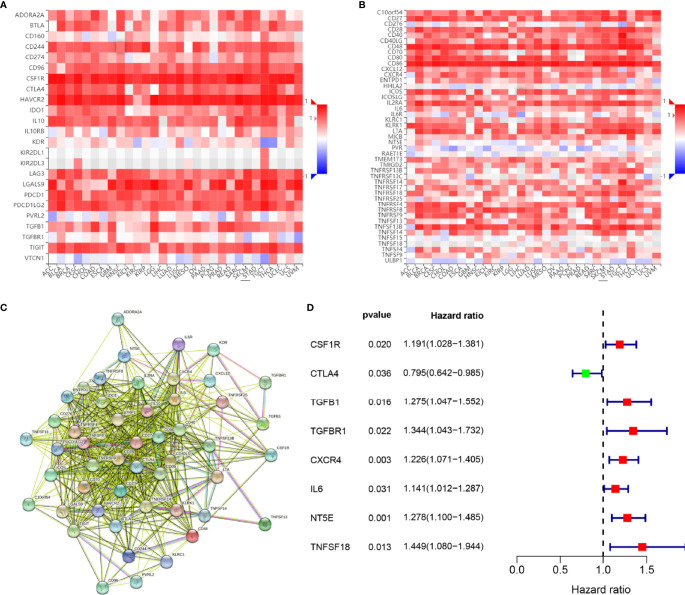
Identification and analysis of immunomodulators associated with the *SPI1* gene. **(A)** Heatmap showing correlations between immunoinhibitors and the *SPI1* gene in STAD. **(B)** Heatmap showing correlations between immunostimulators and the *SPI1* gene in STAD. **(C)** Protein–protein network of 51 SPI1-associated immunomodulators. **(D)** Forest plot showing eight immunomodulators genes strongly associated with GC prognosis identified by the univariate Cox regression method.

**Figure 7:**
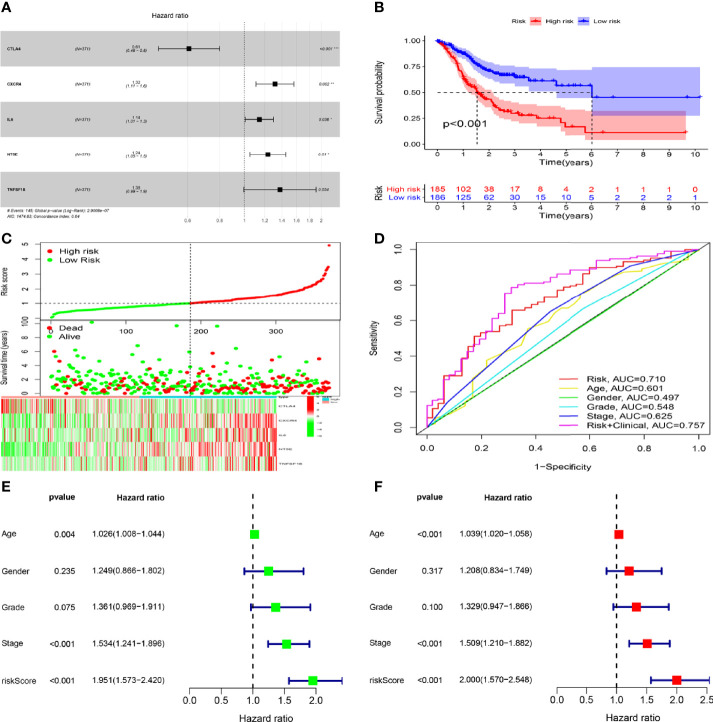
Construction and validation of risk signatures of SPI1-related immunomodulators. **(A)** HRs of genes integrated into the risk signatures are shown in the forest plots for STAD. **(B)** Comparison of Kaplan–Meier curves of the high- and low-risk groups were compared. **(C)** Distribution of risk score, survival status, and gene expression among patients. **(D)** ROC curves showing the predictive efficiency of risk score, age, sex, grade, and stage for 5-year survival rate. **(E, F)** Univariate and multivariate Cox regression analyses of prognostic factors. *P < 0.05, **P < 0.01, ***P < 0.001.

To investigate the prognostic values of SPI1-related immunomodulators in GC, we first used TISIDB to find immunomodulators that were closely related to SPI1. Based on the correlation analysis, we found that many immunomodulators were correlated significantly with the expression of SPI1 in GC (**Figures 6A, B**). After defining the thresholds of P < 0.05 and r > 0.6 to obtain effective immunomodulators, a total of 51 immunomodulators were identified as closely related to SPI1 expression, and a protein–protein interaction network was constructed (**Figure 6C**). We performed univariate Cox regression analysis to further evaluate the relationship between these immunomodulators and the survival of patients with GC. The results showed that eight SPI1-related immunomodulators were closely related to the prognosis of GC patients (**Figure 6D**). We next subjected these immunomodulator genes to stepwise multivariate Cox regression analysis and constructed risk signatures to accurately predict the prognosis of patients with GC (**Figure 7A**). The risk scores were calculated by summing the product of the expression value and coefficient of each gene. Subsequently, all TCGA STAD patients were further divided into high- and low-risk groups, with the median risk score as the cutoff value. The Kaplan–Meier survival curve indicated that patients with low risk scores had significantly longer survival than those with high risk scores (log-rank test, P < 0.001) (**Figure 7B**). In addition, the number of deaths in the high-risk group was significantly higher than that in the low-risk group (**Figure 7C**). The area under the curve (AUC) values for risk score, age, gender, grade, and stage were 0.710, 0.601, 0.497, 0.548, and 0.625 respectively. An AUC of 0.757 was achieved when risk score and clinicopathological factors were combined (**Figure 7D**). Moreover, both univariate and multivariate Cox regression analysis showed that the risk score was an independent prognostic factor in patients with GC (**Figures 7E, F**).

### Construction of Nomogram

**Figure 8:**
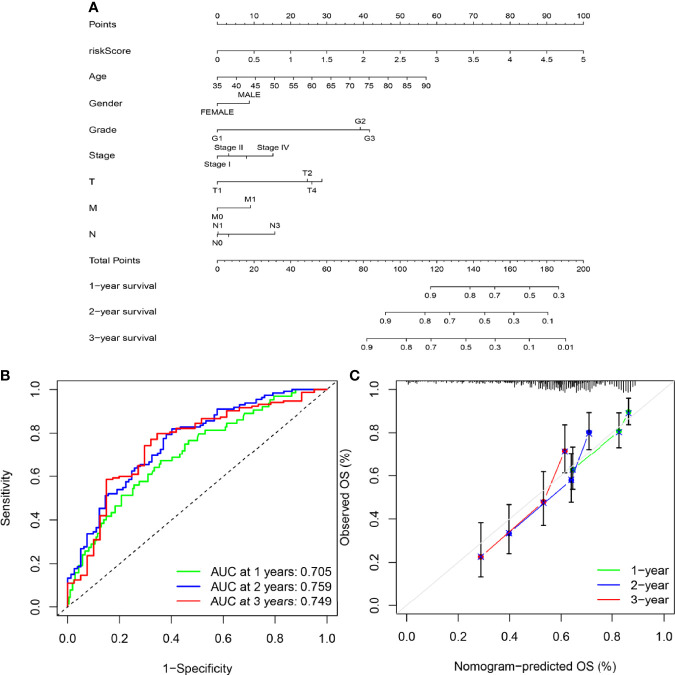
Construction of the nomogram. **(A)** A nomogram was constructed based on independent factors to predict 1- year, 2-year, and 3-year OS in GC patients. **(B)** ROC curve showing the predictive efficiency of the nomogram for 1-year, 2-year and 3-year survival. **(C)** Calibration curve for the prediction of 1-year, 2-year and 3-year survival in GC patients.

To provide a quantitative method with clinical value to predict the probability of 1-, 2- and 3-year OS in GC, we constructed a nomogram by weighting risk score, age, gender, grade, stage, T, N, and M (**Figure 8A**). Total scores were calculated according to the points of all variables in the nomogram; then, the 1-, 2-, and 3-year survival of each patient with GC could be predicted by drawing a vertical line from the total points to the survival prediction axis. The ROC curves showed that The AUC values of 1-year, 2-year and 3-year were 0.705, 0.759 and 0.749 respectively. (**Figure 8B**). The prediction values of the 1-, 2- and 3-year nomograms in the calibration plot were close to the 45-degree line in the complete dataset, which indicates that the nomogram demonstrates good prediction ability (**Figure 8C**).

### Expression of SPI1 Is Upregulated in GC

**Figure 9:**
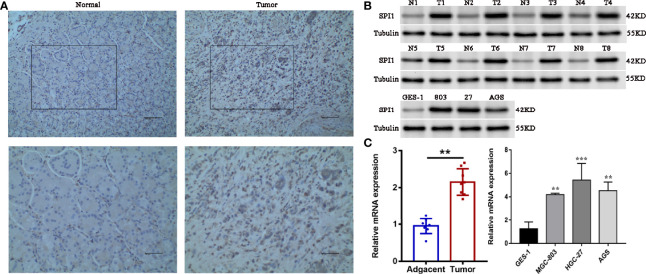
Verification of upregulation of SPI1 expression in GC. **(A)** Immunohistochemical staining of SPI1 in normal and GC tissues (image magnification, x200 and x400). The expression levels of SPI1 in 16 pairs of fresh tumor tissues and adjacent nontumor tissues and GC cell lines were detected by **(B)** western blotting and **(C)** qRT-PCR. **P < 0.01, ***P < 0.001.

To confirm the upregulated expression of SPI1 in GC, a series of experiments were carried out. We performed immunohistochemical staining of SPI1 on clinical specimens of GC. SPI1 showed positive staining mainly in the nucleus in cancerous paraffin sections, whereas negative staining was identified in normal mucosa and the stroma (**Figure 9A**). SPI1 expression was detected in 16 pairs of fresh tumor tissues and adjacent nontumor tissues by western blot and quantitative real-time PCR (qRT-PCR). As shown in **Figures 9B, C**, the protein and RNA levels of SPI1 in GC tissues were significantly higher than those in adjacent nontumor tissues. In addition, SPI1 expression in GES-1 and three different GC cell lines (MGC-803, HGC-27, and AGS) was examined by western blot and qRT-PCR. As shown in **Figures 9B, C**, the protein and RNA levels of SPI1 in GC cell lines were significantly increased compared with those in GES−1 cells.

## Discussion

As a of member of the ETS family of transcription factors, the proto-oncogene *SPI1* was initially considered to be one of the important causative genes in acute leukemia (29, 30). SPI1 plays a significant part in the development, maturation, differentiation, and regulation of myeloid cells and lymphocytes and affects the progression of some diseases. Studies have shown that lower *SPI1* expression can reduce the risk of Alzheimer’s disease by regulating gene expression and cellular function in myeloid lines (monocytes and macrophages) (31). In addition, SPI1 can accelerate the elongation of DNA replication bifurcations without DNA breaks and promote genetic instability; this is linked to its carcinogenicity (32). Ueno et al. found that SPI1 could induce growth arrest and apoptosis of myeloma cells by regulating the expression of certain cell cycle and apoptotic genes (33). Recent studies have also confirmed the prognostic potential of SPI1 in a variety of solid tumors other than hematological malignancies (13–16). Thus, we wondered whether SPI1 could be used as a sensitive biomarker of GC. As far as we know, this is the first study to emphasize the role of SPI1 not only as an immunomodulatory factor but also as an immune-infiltrating protein in GC.

First, we analyzed the expression levels of *SPI1* mRNA in different types of cancers. The results showed that *SPI1* mRNA expression was upregulated significantly in GC and certain other tumors, whereas it was downregulated in colon cancer, lung cancer, and leukemia; this difference may have been caused by the different potential pathogenic mechanisms involved in these cancers. Analysis using Kaplan–Meier Plotter data showed that high expression of *SPI1* was closely related to poor prognosis of GC patients. High *SPI1* expression was correlated with high HR for poor OS, PFS, and PPS in GC. Having shown that SPI1 could affect the prognosis of patients with GC, we further analyzed the correlations between *SPI1* expression and different stages of GC and found that the expression of *SPI1* in advanced GC was much higher than that in early GC.

To confirm the upregulated expression of SPI1 in GC, a series of experiments were carried out. The results showed that the mRNA and protein levels of SPI1 were significantly upregulated in GC tissues and cell lines. Overall, these findings provide strong support for SPI1 as a diagnostic and prognostic biomarker for GC.

In addition, studies have shown that CNAs could lead to abnormal gene expression, resulting in a variety of genetic diseases. For example, heterozygous deletion of the *SPI1* locus can lead to the occurrence of AML (10). To explore the possible reasons for the increased expression of *SPI1* in GC, we analyzed *SPI1* gene expression and genomic alterations in GC. The results showed that *SPI1* expression was increased significantly in the diploid group, and that *SPI1* expression was related significantly to *SPI1* diploidy in GC. This suggested that diploidy might be one of the mechanisms of upregulation of SPI1 in GC. However, there was a significant negative correlation between *SPI1* expression and methylation (HM450), which was also consistent with previous reports (34).

Many articles have reported that TICs can induce an immune microenvironment in GC, thus affecting the prognosis of patients with GC. According to CIBERSORT analysis of the proportions of TICs, activated memory CD4 T cells, M2 macrophages, resting NK cells, and resting dendritic cells were correlated positively with SPI1 expression in patients with GC. We believed that SPI1 in GC can specifically recruit some immune cells to gather at the tumor site, thereby changing the proportions of infiltrating immune cells. With the deepening of research, more and more researchers have found that some immune cells are closely related to the occurrence, progression, metastasis and prognosis of tumors (35–37). Studies have shown that increased levels of intratumoral CD4 T cells are associated with tumor progression and predict poorer patient survival in GC (38). Chen et al. found that M2 macrophages could secrete chitinase 3 like 1 and activate downstream pathways to promote the metastasis of GC cells and breast cancer cells *in vivo* and *in vitro* (39). In addition, Lan et al. also found that M2 macrophages can induce the migration and invasion of colorectal cancer cells through M2 macrophage-derived exosomes (40). Therefore, we have reason to believe that SPI1, as a key regulator of immune signal transduction, can promote the progression of gastric cancer by regulating the composition and proportion of tumor infiltrating immune cells. In addition, our study demonstrated that SPI1 diploidy was significantly related to the levels of immune infiltration of different types of immune cells in GC. To sum up, our research suggested that SPI1 may be a new target for immunotherapy.

To further determine the biological functions of SPI1 in the development of GC, we performed GO and KEGG enrichment analyses. The results suggested that SPI1 might exert its biological effects on the progression of GC *via* regulation of immunobiological processes, the cell cycle, and DNA replication. GO analysis showed enrichment of critical immune biological processes, including T cell activation, regulation of lymphocyte activation, leukocyte cell–cell adhesion, and signaling pattern recognition receptor activity. This partly explains the role of SPI1 in the activation of CD4 T memory cell and M2 macrophages that was suggested by the CIBERSORT analysis. Hosokawa et al. revealed that the transcription factor SPI1 regulated gene expression in early T cell development by recruiting partner transcription factors to its own binding sites (41). The growth and development of macrophages was also inseparable from the normal expression of the *SPI1* gene (8). These results further supported the hypothesis that SPI1 promoted the progression of GC by influencing the level of immune infiltration through immune activation. Furthermore, the results of KEGG enrichment pathway analysis showed that the cell cycle, spliceosome, and RNA transport pathways were enriched. Our results are consistent thus with previous reports that SPI1 can regulate the cell cycle and apoptosis (11, 12). Apoptosis is also a potential mechanism for cancer. Defects in apoptosis-related pathways lead to malignant transformation, tumor metastasis, and drug resistance of the affected cells (42). Thus, SPI1 and its co-expressed genes might affect the prognosis of patients with GC *via* participating in the cell cycle and apoptosis. It was the regulatory effect of SPI1 on immune infiltration and cell cycle that greatly promoted the progression of GC and led to a poor prognosis of patients with GC.

Increasing numbers of studies have shown that signals based on specific gene expression can accurately predict the prognosis of patients with GC (43–45). As previous studies have shown that SPI1 can be used as a key regulator of signal communication in the immune system and is associated with poor prognosis in patients with GC, we investigated whether a signature based on SPI1-related immunomodulators could accurately predict the prognosis of patients. SPI1-related immunomodulators were constructed based on immunoinhibitors and immunostimulators that were significantly correlated with SPI1 at the gene expression level (P<0.05, r > 0.6). The results of ROC curve showed that risk signature had the highest accuracy in predicting survival compared with other clinical features.Independent prognostic analysis showed that the risk signature can be used as an independent prognostic determinant of patients with GC. In addition, a nomogram was constructed based on the risk signature and other independent clinical features to predict the 1-, 2- and 3-year overall survival rates of individual patients with GC. The results of the ROC curve and the calibration curve showed that the nomogram we constructed can provide clinicians with a more accurate, convenient and practical prediction tool.

## Conclusions

In conclusion, we found that SPI1 expression was upregulated in GC, and was related to poor prognosis and disease progression. SPI1 might promote tumor progression *via* regulating immune infiltration and the cell cycle of GC. In addition, the risk signature based on SPI1-related immunomodulators can guide clinicians to judge the prognosis of patients with GC. Thus, SPI1 can be identified a diagnostic and prognostic biomarker and immune-related therapeutic target for GC. However, further studies are needed to confirm these results and reveal the underlying mechanisms.

## Data Availability Statement

The original contributions presented in the study are included in the article/**Supplementary Material**. Further inquiries can be directed to the corresponding authors.

## Author Contributions

MJ and ZJ conceived and designed the experiments. JH and WC analyzed the data and wrote the manuscript. All authors read and approved the final manuscript.

## Funding

This work was supported by the National Natural Science Foundation of China (No.81960503), Postgraduate Innovation Special Foundation of Jiangxi Province (YC2020-B054).

## Conflict of Interest

The authors declare that the research was conducted in the absence of any commercial or financial relationships that could be construed as a potential conflict of interest.

## Publisher’s Note

All claims expressed in this article are solely those of the authors and do not necessarily represent those of their affiliated organizations, or those of the publisher, the editors and the reviewers. Any product that may be evaluated in this article, or claim that may be made by its manufacturer, is not guaranteed or endorsed by the publisher.

## Supplementary Material

Supplementary Figure 1 Correlation analysis of infiltrating immune cells in tumor specimens. **(A)** Stacked bar chart showing the proportion of 22 kinds of immune cells in STAD tumor samples. **(B)** Correlation matrix of 22 kinds of immune cell proportions. **(C)** Violin plot displaying the differentially infiltrated immune cells between STAD tumor samples with high or low SPI1 expression relative to the median SPI1 expression level. Blue represents the SPI1-low expression group and red represents the SPI1-high expression group.

Supplementary Figure 2 KEGG pathway annotations of cell adhesion molecules. Red denotes leading edge genes; green denotes the remaining genes.

Supplementary Figure 3 KEGG pathway annotations of the cell cycle pathway. Red denotes leading edge genes; green denotes the remaining genes.
