# Supplementary figures and images for: Comprehensive Analysis of Immune Implications and Prognostic Value of SPI1 in Gastric Cancer
Source: Front Oncol. 2022 Feb 14;12:820568. doi: 10.3389/fonc.2022.820568 (PMC8882873; doi:10.3389/fonc.2022.820568)

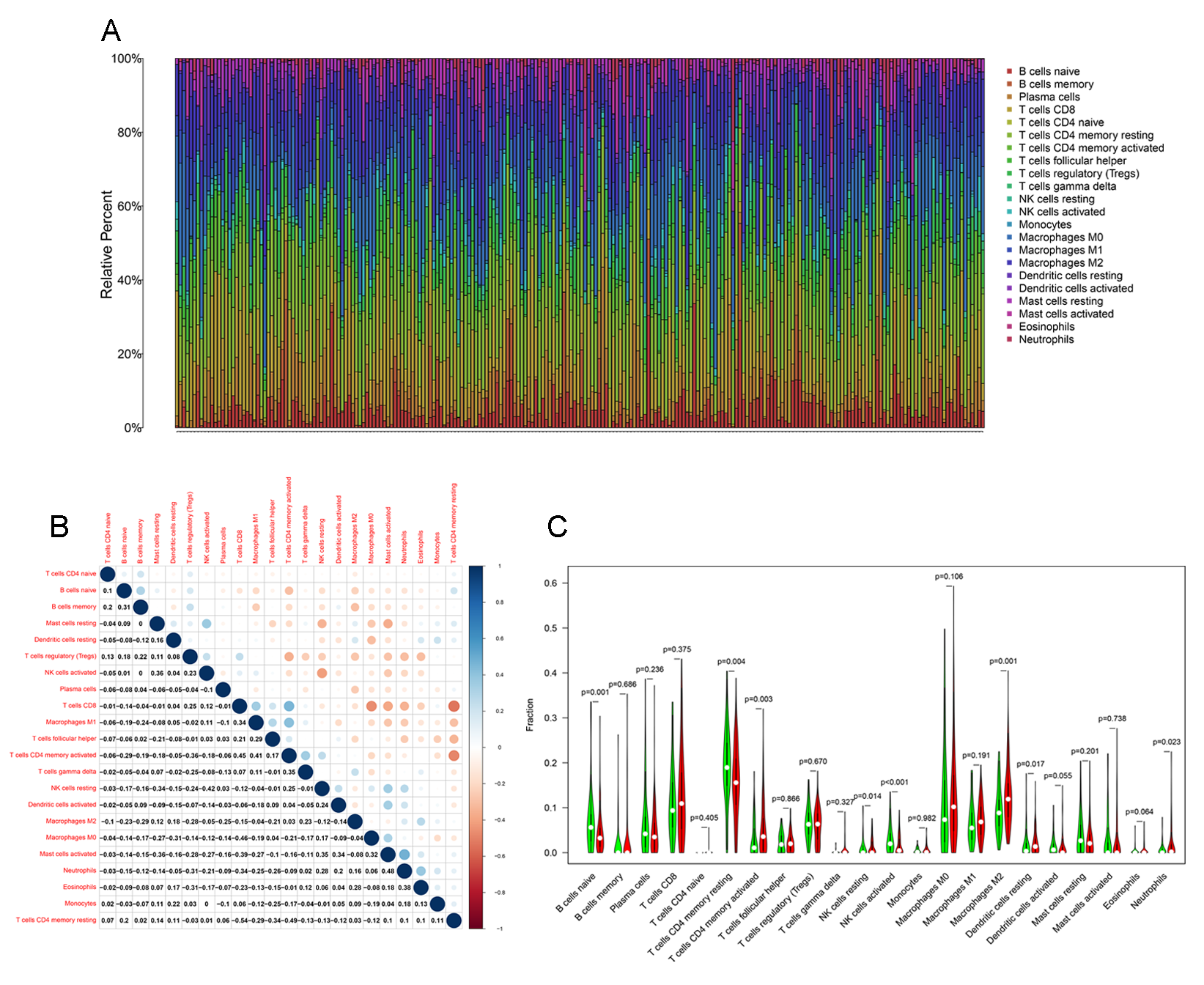

Supplement: Supplementary Figure 1 — Correlation analysis of infiltrating immune cells in tumor specimens. (A) Stacked bar chart showing the proportion of 22 kinds of immune cells in STAD tumor samples. (B) Correlation matrix of 22 kinds of immune cell proportions. (C) Violin plot displaying the differentially infiltrated immune cells between STAD tumor samples with high or low SPI1 expression relative to the median SPI1 expression level. Blue represents the SPI1-low expression group and red represents the SPI1-high expression group. [file Image_1.tif]

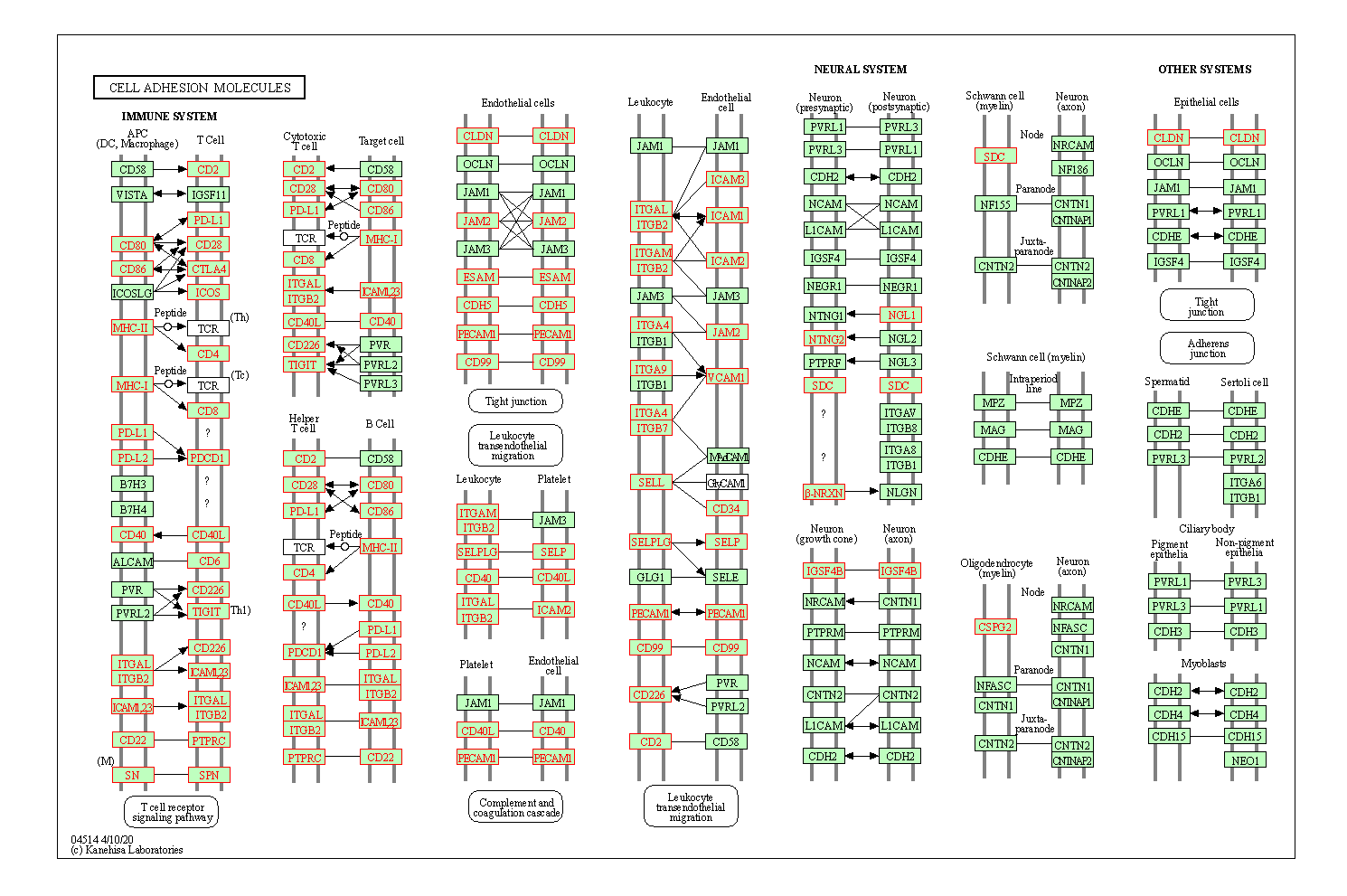

Supplement: Supplementary Figure 2 — KEGG pathway annotations of cell adhesion molecules. Red denotes leading edge genes; green denotes the remaining genes. [file Image_2.tif]

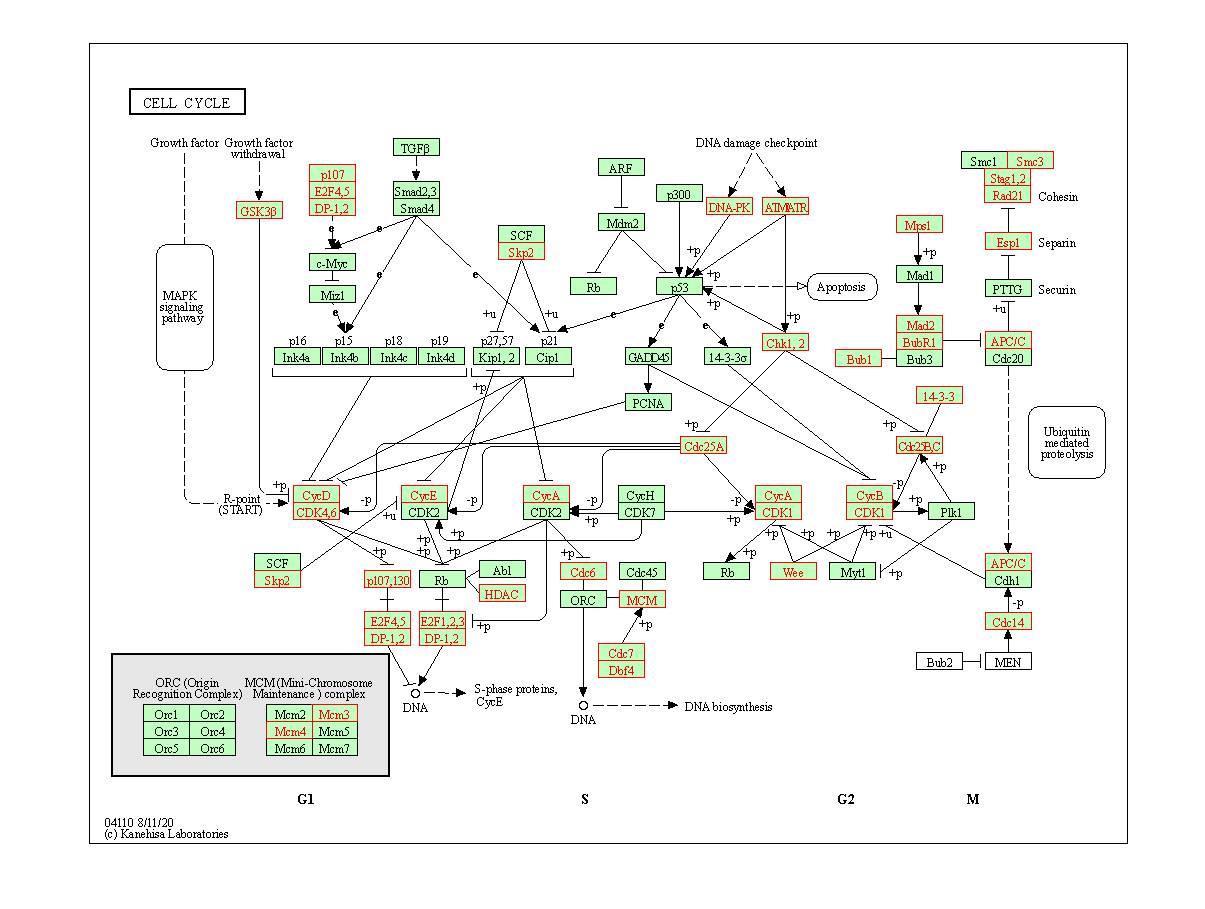

Supplement: Supplementary Figure 3 — KEGG pathway annotations of the cell cycle pathway. Red denotes leading edge genes; green denotes the remaining genes. [file Image_3.tif]
